# Experimental and quantitative imaging techniques in interstitial lung disease

**DOI:** 10.1136/thoraxjnl-2018-211779

**Published:** 2019-03-18

**Authors:** Nicholas D Weatherley, James A Eaden, Neil J Stewart, Brian J Bartholmai, Andrew J Swift, Stephen Mark Bianchi, Jim M Wild

**Affiliations:** 1 Academic Unit of Academic Radiology, University of Sheffield, Sheffield, UK; 2 Department of Radiology, Mayo Clinic Minnesota, Rochester, Minnesota, USA; 3 Department of Respiratory Medicine, Sheffield Teaching Hospitals Foundation Trust, Sheffield, UK

**Keywords:** interstitial fibrosis, imaging/ct mri etc, drug induced lung disease, idiopathic pulmonary fibrosis

## Abstract

Interstitial lung diseases (ILDs) are a heterogeneous group of conditions, with a wide and complex variety of imaging features. Difficulty in monitoring, treating and exploring novel therapies for these conditions is in part due to the lack of robust, readily available biomarkers. Radiological studies are vital in the assessment and follow-up of ILD, but currently CT analysis in clinical practice is qualitative and therefore somewhat subjective. In this article, we report on the role of novel and quantitative imaging techniques across a range of imaging modalities in ILD and consider how they may be applied in the assessment and understanding of ILD. We critically appraised evidence found from searches of Ovid online, PubMed and the TRIP database for novel and quantitative imaging studies in ILD. Recent studies have explored the capability of texture-based lung parenchymal analysis in accurately quantifying several ILD features. Newer techniques are helping to overcome the challenges inherent to such approaches, in particular distinguishing peripheral reticulation of lung parenchyma from pleura and accurately identifying the complex density patterns that accompany honeycombing. Robust and validated texture-based analysis may remove the subjectivity that is inherent to qualitative reporting and allow greater objective measurements of change over time. In addition to lung parenchymal feature quantification, pulmonary vessel volume analysis on CT has demonstrated prognostic value in two retrospective analyses and may be a sign of vascular changes in ILD which, to date, have been difficult to quantify in the absence of overt pulmonary hypertension. Novel applications of existing imaging techniques, such as hyperpolarised gas MRI and positron emission tomography (PET), show promise in combining structural and functional information. Although structural imaging of lung tissue is inherently challenging in terms of conventional proton MRI techniques, inroads are being made with ultrashort echo time, and dynamic contrast-enhanced MRI may be used for lung perfusion assessment. In addition, inhaled hyperpolarised ^129^Xenon gas MRI may provide multifunctional imaging metrics, including assessment of ventilation, intra-acinar gas diffusion and alveolar-capillary diffusion. PET has demonstrated high standard uptake values (SUVs) of ^18^F-fluorodeoxyglucose in fibrosed lung tissue, challenging the assumption that these are ‘burned out’ and metabolically inactive regions. Regions that appear structurally normal also appear to have higher SUV, warranting further exploration with future longitudinal studies to assess if this precedes future regions of macroscopic structural change. Given the subtleties involved in diagnosing, assessing and predicting future deterioration in many forms of ILD, multimodal quantitative lung structure-function imaging may provide the means of identifying novel, sensitive and clinically applicable imaging markers of disease. Such imaging metrics may provide mechanistic and phenotypic information that can help direct appropriate personalised therapy, can be used to predict outcomes and could potentially be more sensitive and specific than global pulmonary function testing. Quantitative assessment may objectively assess subtle change in character or extent of disease that can assist in efficacy of antifibrotic therapy or detecting early changes of potentially pneumotoxic drugs involved in early intervention studies.

## Introduction

Imaging plays a key role in the diagnosis and assessment of interstitial lung disease (ILD). A multidisciplinary team with expertise in ILD can often reach a reliable diagnosis based on clinical findings and radiology alone, as exemplified by the Joint Consensus International Societies Statement on the classification of idiopathic interstitial pneumonias.^1^ In practice, radiological examinations are qualitatively interpreted with an inherent degree of subjectivity. However, ILD features such as honeycombing are often subtle and may be mimicked by other conditions, leading to interobserver disagreement in their presence and extent.^2^ Thus, interest exists in developing quantitative and novel imaging tools. We provide a commentary on the recent developments in novel imaging techniques in ILD, with a focus on quantitative high-resolution CT (HRCT) and new techniques from MRI and positron emission tomography (PET).

## Methods

Literature searches were performed using the Medline database via Ovid online portal at the University of Sheffield (UK) and cross-referenced with identical search terms on PubMed and TRIP database (both web based and open access). Two reviewers (NDW and JAE) independently performed the search and identified articles for inclusion. Only human studies since 1998 with n>1 were included. Studies were assessed on methodological approach, bias and quality using the Scottish Intercollegiate Guidelines Network (SIGN) checklist. In case of disagreement about inclusion, an arbitrator (JMW) made the final decision and acted as guarantor. The Preferred Reporting Items for Systematic Reviews and Meta-Analyses flow diagram and search terms are provided in two online supplements.

## Quantitative imaging

### Computed tomography

HRCT is a highly sensitive imaging tool for the assessment of macrostructural changes in ILD. In the era of multidetector CT scanners, volumetric protocols acquired during inspiration are preferred to non-contiguous slices due to enhanced sensitivity to spatially heterogeneous ILD features.^3^ HRCT plays a key role in identification of the pathological phenotype of ILD, and typical imaging features are well recognised in international consensus guidelines.^1^ Even so, disagreement on presence or quantification of disease severity between independent radiologists is common.^2 4 5^ Semiquantitative CT scoring analysis by expert radiologists may provide prognostic insight,^6^ but there remains no standard method for scoring radiological extent or severity. Quantification of overall lung histogram features, regional CT density changes, parenchymal texture features and other assessments by advanced algorithms including unsupervised machine learning and deep learning approaches to image analysis have the potential to standardise and develop the role of HRCT in ILD.

### Lung density analysis

HRCT density measurements have been used to quantitatively assess lung structure in a range of respiratory conditions, most prominently in emphysema.^7^ The CT density histogram of normal lung tissue is peaked at approximately −800 Hounsfield units and is left-skewed. An increase in the amount of soft tissue, due to fibrosis, will increase mean lung density (MLD) and decrease the histogram kurtosis and skew.^8^ Whole-lung CT metrics, such as MLD,^8^ or lowest fifth percentile of the lung density histogram,^9^ correlate with physiological measures of severity, and change with disease progression.^10^ Although several studies have explored the relationship between density histogram metrics and ILD outcomes,^11–14^ reducing quantitative regional information from highly sensitive imaging to global summary measurements sacrifices the richness of the imaging data. Global measures are also confounded by other features such as air trapping in hypersensitivity pneumonitis (HP) and parenchymal destruction in combined pulmonary fibrosis and emphysema (CPFE) syndrome.

The ability of lung density analysis to differentiate between usual interstitial pneumonia (UIP) pattern fibrosis/idiopathic pulmonary fibrosis (IPF) and other types of ILD is debatable. Do *et al* demonstrated significant differences in kurtosis and skewness between 28 patients with non-specific interstitial pneumonia (NSIP) and 32 patients with UIP pattern of fibrosis.^15^ However, two studies by Sverzellati *et al* did not identify a significant difference in kurtosis, skewness and mean lung attenuation between UIP pattern fibrosis/IPF patients and patients with HP and/or unclassifiable idiopathic interstitial pneumonia.^16 17^

### Texture-based analysis and machine learning

Texture-based analysis and computer vision-based approaches can be applied to imaging data to characterise, model and process imaging features, simulating human visual perceptual and learning processes using two dimensional or volumetric classification algorithms. Focal texture analysis can potentially evaluate both global and regional information, generating quantitative metrics. The texture features can allow for both density and morphological assessment that can provide a means of determining the type of abnormality (such as emphysema vs honeycombing vs cysts), the severity (fine vs coarse reticulation) and extent of disease that correlates with expert radiologist assessment.^18^

The adaptive multiple feature method (AMFM) is a lung texture analysis software that has been designed to recognise HRCT patterns. AMFM uses a combination of 26 different mathematical features describing regional density patterns, along with a Bayesian classifier to recognise and quantify the volume occupied by a variety of radiological patterns. This can distinguish emphysema from fibrosis, identify ground glass opacification (GGO) and normal lung.^19–21^ The two-dimensional AMFM appeared relatively unsuccessful in identifying honeycombing^19 20^ due to the complex appearance of high and low attenuation values in these regions.^22^ Further work on the AMFM allowed three-dimensional (3D) assessment.^23^ Sensitivity and specificity for automated identification was 100% for emphysema and consolidation and 95% and 97%, respectively, for honeycombing.^23^ AMFM was retrospectively applied to the HRCT scans in a large clinical trial of patients with IPF, where extent of GGO was independently associated with disease progression.^21^ Boehm and colleagues demonstrated that combining densitometric and topological information allowed automated analysis to reproduce radiologists’ ratings on disease severity, ranging from 85.7% agreement in fibrotic regions to 98% in normal regions.^24^

Asakura *et al* created the Gaussian Histogram Normalized Correlation (GHNC) system, which uses local histograms and the degree of CT attenuation to separate the lungs into five categories.^25^ Using GHNC, Iwasawa *et al* showed a smaller increase in fibrosis score and F-pattern volume on the follow-up CTs in 38 patients with IPF treated with pirfenidone compared with 40 age-matched controls with IPF.^5^ There were no significant differences in the sensitivity, specificity and accuracy between GHNC analysis, visual CT score and radiologist interpretation.

Defining lung regions manually is time consuming, therefore automated segmentation of lung fields may allow more widespread use of quantitative CT. Pathology adjacent to the pleural surface can be problematic, as ILD features may be similar in appearance to soft tissue.^10 26^ Korfiatis *et al* described how a support vector machine can be used to perform an iterative ‘neighbourhood labelling’ process, whereby lung voxels in the border regions of the lung are iteratively rechecked against labelled voxels to refine the lung border, thus improving delineation of lung tissue.^27^

Unsupervised feature leaning paradigms, such as data-driven textural analysis (DTA), are able to use clustering analyses to find common features from a collection of raw images. Humphries *et al* used the DTA algorithm to analyse 55 CT scans from the IPFnet ACE study, identifying consistent low-level pixel patterns and thus generating a ‘dictionary’ of ILD elements. These were compared with 35 CTs from the COPDGene study as examples of non-fibrotic lungs. The derived fibrosis score (fibrotic regions of interest (ROIs)/total ROI) correlated with pulmonary function tests (PFTs) in 280 patients with IPF at baseline and decrease in FVC at follow-up was associated with an increase in DTA fibrosis score in 72 follow-up patients.^28^

**Figure 1:**
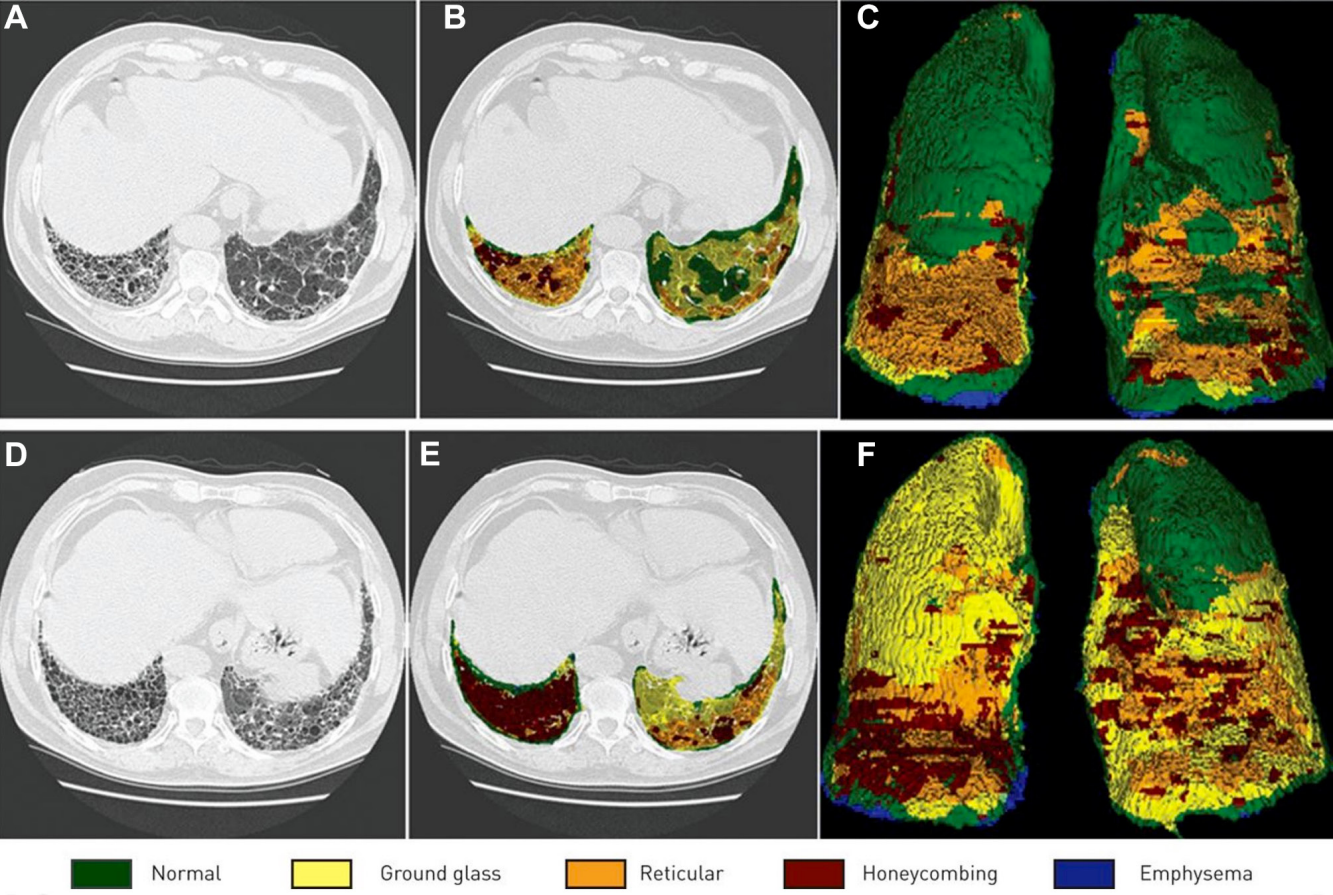
Representative results from the quantitative analysis of CALIPER. Each row (A–C) and (D–F) correspond to the two timepoints from one patient with usual interstitial pneumonia. The individual voxels of the lung regions in the original sections (A and D) are classified and colour coded into one of the classes of visible abnormalities (B and E). Three-dimensional maximum feature projections are also shown (C and F). Reproduced with permission from European Respiratory Society.^33^ CALIPER, Computer Aided Lung Informatics for Pathology Evaluation and Rating.

Computer Aided Lung Informatics for Pathology Evaluation and Rating (CALIPER) is an image analysis tool that uses both 3D histogram features within a regional voxel and morphological analysis to characterise HRCT data. The classifier was developed based on consensus radiologist determination of parenchymal features of voxels randomly selected from training images with histopathologically confirmed disease for a variety of lung parenchymal pathologies and control subjects^29–31^ from the Lung Tissue Research Consortium.^32^ In retrospective clinical assessment of 55 patients with IPF, CALIPER-measured ILD changes including percent ILD, total ILD volume and total reticulation volume were associated with survival at multivariate analysis.^33^ An example output from CALIPER in a patient with UIP is shown in figure 1.

## Vessel quantification

Jacob *et al* report automatic segmentation of the pulmonary vasculature, defining quantitative output in terms of vessel cross sectional area.^34 35^ The sum of pulmonary vessel volume (PVV) not only correlated with visually and quantitatively assessed extent of disease, but when combined with physiological parameters, prognosticated better than the Gender, Age, and Physiology (GAP) score.^34^ In 203 patients with connective tissue disease ILD (CTD-ILD), PVV outperformed physiological indices and visual CT scores as predictors of mortality.^35^ In IPF, upper zone CALIPER vessel-related structure variables were found to be the strongest predictors of death and/or 10% FVC decline at 12 months.^36^

As the volumetric assessment of the size and morphology of branching vascular structures is extremely difficult for a radiologist, this novel metric highlights the potential utility of quantitative measures that do not have a current visual or morphological correlate in the radiology lexicon. Additional machine learning or deep learning techniques may elucidate features that are not perceptible nor reproducibly assessed by humans.

## Limitations

Several issues exist in the standardisation of quantitative CT metrics. Multicentre trials are confounded by differences in scanner equipment and protocols. Choice of reconstruction algorithm influences image resolution and density histogram parameters. In particular, a high-frequency reconstruction algorithm reduces per pixel histogram skewness and kurtosis.^37^ Regular quality assurance by scanning phantoms at all collaborating sites is not always feasible.^11^ Standardisation of the image density to tracheal air density is plausible,^38^ but density varies throughout the trachea. Chong *et al* have reported on a novel feature selection scheme that prioritises recognition features robust to variations in apparent lung density, reporting enhanced performance in correctly identifying key disease features in ILD.^39^ In spite of such obstacles, Iwisawa and colleagues reported excellent agreement in automated CT pathology features across different scanners and sites using the GHNC analysis technique.^40^

Lack of standardisation of inspiration level during CT acquisition may also lead to differences in density. Previous data suggests inspiration to a lung volume 90% of vital capacity yields the most reproducible density results.^41^ However, spirometric gating is not routinely available in most centres, and some researchers report adequate reproducibility without spirometric standardisation of inspiratory effort.^12^ Comorbidities such as heart failure or exogenous factors such as contrast media are likely to affect the overall lung density, but quantitative analysis of their impact is lacking.

## CT summary

Recent significant advances in quantitative CT have been made in semiautomated lung segmentation and greater success in the automated identification of honeycombing using texture analysis, alongside new means of assessing the associated pulmonary vascular changes. While global physiological indices of disease progression such as PFTs are affected by comorbidities such as emphysema, ideally quantitative CT should objectively and accurately measure structural interstitial change as a standalone parameter, enabling effective analysis of treatment response or objective assessment of disease progression. At present, these methods require varying degrees of manual input, which will need to be automated before rolling out the technology for use in routine clinical practice.

### Magnetic resonance imaging

Acquisition of lung MRI is challenging due to a number of factors, including low proton density of lung tissue, multiple air tissue interfaces causing magnetic field inhomogeneity, rapid MR signal relaxation and respiratory and cardiac motion. However, proton MRI methods such as ultrashort echo time (UTE) offer the possibility to image ILD structural changes at greater resolution, and contrast-enhanced imaging offers a means of pulmonary perfusion assessment. In addition, MRI of inhaled hyperpolarised gases offer the ability to assess changes in lung ventilation,^42^ microstructure^43 44^ and gas exchange assessment.^45^

## Proton density, T1 and T2 MRI

**Figure 2:**
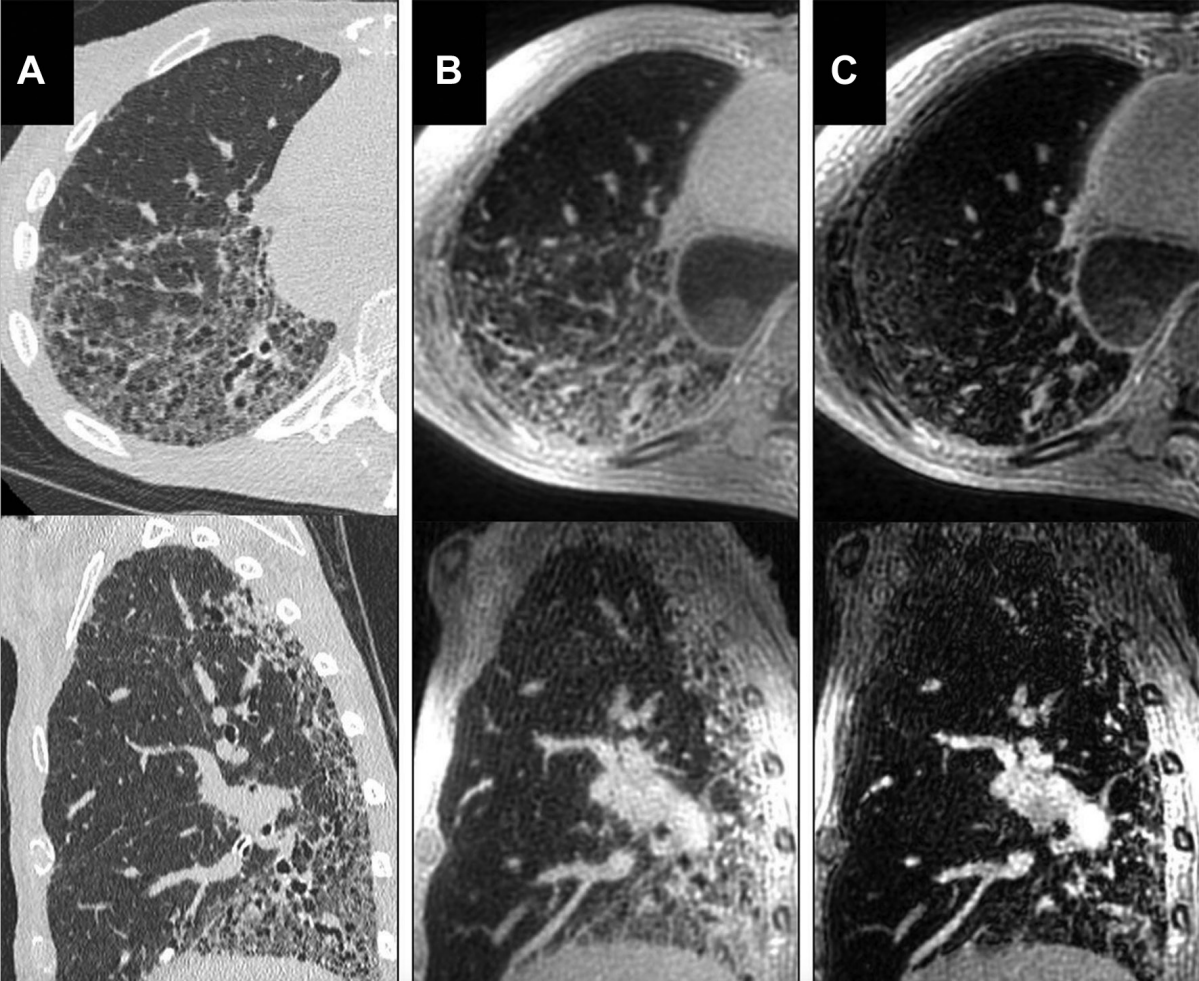
Axial and sagittal reformats of the high-resolution CT (HRCT) (A), three-dimensional ultrashort echo time (3D UTE) (B) and the second echo of the 3D UTE sequence (C). Although honeycomb change is visualised in the UTE image, HRCT images show higher spatial resolution. The structural abnormality is not well visualised in the second echo images. Reproduced with permission from John Wiley and Sons.^49^

Established proton MRI techniques such as 3D gradient echo and balanced steady-state free precession demonstrate reasonable sensitivity to fibrotic regions of lung tissue, although qualitative structural assessment is undoubtedly inferior to CT.^46^ Subtle abnormalities such as thickening of lobar septae are difficult to appreciate on MRI.^47^ In a retrospective study of CT and MRI in systemic sclerosis (SSc) associated ILD, extent of disease on MRI and CT correlated well, but MRI underscored more extensive disease.^48^ More recently, UTE imaging with radial acquisition of k-space, such as that shown in figure 2, has enabled reductions of echo time to less than 100 μs, thus minimising T2* dephasing signal losses resultant from magnetic field inhomogeneity at air–tissue interfaces. With 3D free breathing acquisition, these methods provide improved structural image quality and resolution at the cost of motion artefact at the lung bases and long acquisition time, as demonstrated in figure 2.^49^ With the introduction of UTE MRI, the diagnostic accuracy of proton MRI in ILD has been found to be comparable with that of HRCT.^48 50 51^

Regional T1 and T2 relaxation time characteristics of lung tissue may discriminate ILD pathology. Stadler *et al* demonstrated that the T1 of fibrotic lung parenchyma is significantly longer than emphysema, but these T1 values were heavily influenced by lung inflation state.^52^ Buzan *et al* observed that GGO, reticulation and honeycombing have inherently different T2 relaxation times in 12 patients with NSIP or UIP.^53^ A larger study by the same author published 2 years later confirmed a strong positive correlation between T2 relaxation and proton density in NSIP (r=0.64, p<0.001), but this correlation was weak in UIP pattern of fibrosis (r=0.20, p=0.01).^54^ In the NSIP group, those with suspected inflammatory activity had statistically significant increased T2 relaxation times when compared with those with suspected stable disease. Unfortunately, the difference in T2 relaxation times between UIP and NSIP was not statistically significant, thereby suggesting that T2-weighted proton MRI is not a reliable method to differentiate between these two important patterns of pulmonary fibrosis.

MR elastography (MRE) is a method of measuring tissue stiffness by measuring acoustic shear wave propagation. A recent study by Marinelli *et al* used MRE to quantify a difference in topographical distribution of shear stiffness between 15 patients with ILD (including eight with IPF) compared with 11 healthy volunteers.^55^ They found that with increasing transpulmonary pressure (from residual volume to total lung capacity), the lung stiffness increased. It would be interesting to see if future studies using MRE in ILD can demonstrate this as a valuable diagnostic tool in monitoring of disease progression and providing dynamic lung function data.

### Oxygen-enhanced proton MRI

Oxygen-enhanced MRI uses the paramagnetic (T1-shortening) effect when molecular oxygen dissolves in tissue water and blood in the lungs. By acquiring images using a paradigm of alternating inhaled oxygen at room air concentration and high flow oxygen and thereafter subtracting the images, maps of T1-shortening are derived. Müller *et al* found a statistically significant difference in the signal intensity (SI) changes and SI slopes between 17 patients with various pulmonary diseases (including 12 with IPF and 1 with HP) and 11 healthy volunteers.^56^ A strong correlation was seen between the SI slope values and diffusing capacity of the lungs for carbon monoxide (DLCO), but the correlation was weak between SI change values and DLCO for the section selective inversion pulse. Ohno *et al* demonstrated enhancement changes in patients with CTD-ILD versus healthy controls.^57^ Molinari *et al* used ‘percentage of oxygen-activated pixels’ (OAP%) as a quantitative metric, demonstrating a correlation of OAP% with DLCO in a cohort of patients with various forms of ILD.^58^ Given that the oxygen environment is dependent on both local ventilation and perfusion, separating the contribution of each is a significant current challenge.

### Hyperpolarised gas MRI

Hyperpolarised gas MRI exploits the signal enhancement available by the technique of spin exchange optical pumping of helium (^3^He) or xenon (^129^Xe). These atoms have spin ½ and can thus be imaged with conventional MRI methods with dedicated radio frequency coils.^59^ The hyperpolarised gas is prepared in a pure state or mixed with nitrogen or oxygen and inhaled by the patient, who holds their breath for several seconds during acquisition. This technique is reportedly well tolerated in patients with various lung diseases.^60^

**Figure 3:**
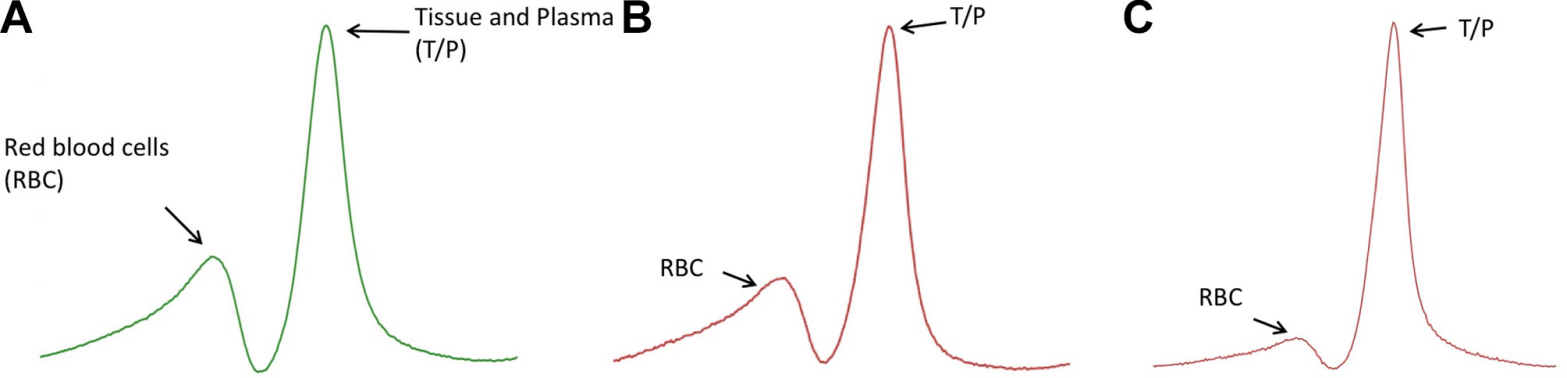
Example of whole-lung spectral peaks generated from hyperpolarised xenon magnetic resonance spectroscopy. Figure part A is generated from a healthy volunteer. The red blood cell (RBC) peak is relatively preserved. In mild (B) and severe (C) IPF, with gender, age, and physiology (GAP) scores 1 and 3, respectively, the RBC peak is diminished with respect to the tissue/plasma peak, suggesting a diminishment in gas transfer efficiency of the lung.

**Figure 4:**
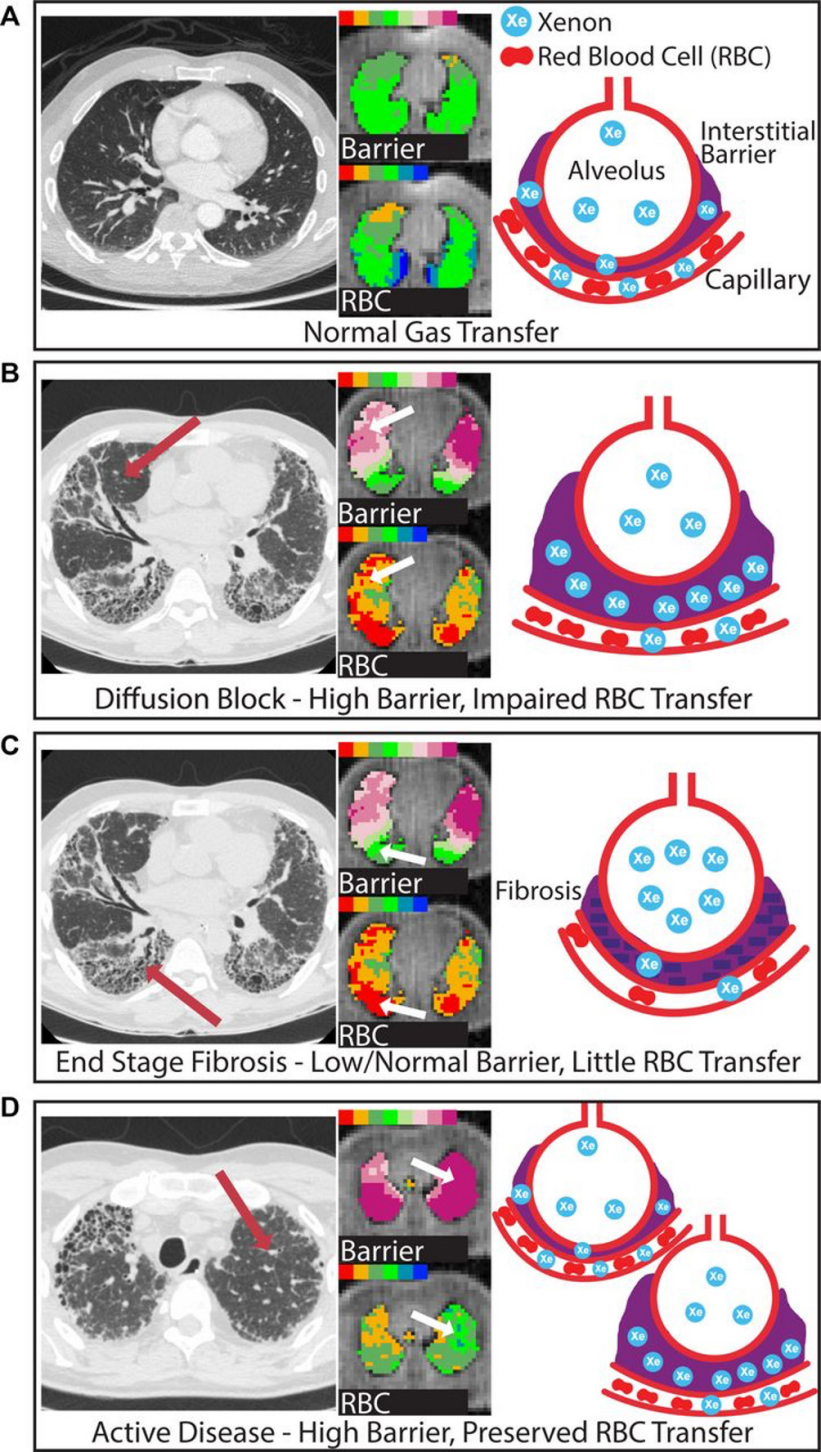
Wang *et al* demonstrated how parametric maps derived from hyperpolarised xenon (^129^Xe) magnetic resonance spectroscopy may phenotype gas exchange limitation in the lung in idiopathic pulmonary fibrosis (IPF). In healthy lungs (A), xenon gas diffuses efficiently across the alveolar membrane into red blood cells (RBCs), resulting in signal intensities in the normal range for both compartments. In IPF, the interstitium is thickened, increasing xenon uptake in the tissue. In some regions (B, arrows), diffusion across it is slowed, causing RBC transfer to decrease. As the disease progresses (C), scarring becomes so severe that ^129^Xe no longer diffuses into or through the barrier. The authors postulated that regions depicting the coexistence of increased barrier uptake with preserved RBC transfer (D, arrow) may represent regions of early disease. Reproduced with permission from BMJ Publishing Group.^64^

While ^3^He is insoluble in lung tissue, ^129^Xe does not remain exclusively in the airways but crosses the alveolar interstitium into capillary blood. Diffusion limitation can be probed with MR spectroscopic techniques, which take advantage of the unique chemical shift of ^129^Xe in gaseous, aqueous (tissue and plasma [TP]) and red blood cell (RBC) environments. The RBC peak signal was reduced in comparison with the TP signal in patients with IPF versus healthy volunteers, suggesting that spectroscopic approaches may provide a viable biomarker of interstitial thickening.^45^ Indeed, measures of ^129^Xe gas exchange correlated with DLCO in two small cohorts of patients with IPF and SSc-associated ILD.^45 61^ These spectroscopic techniques can be extended to provide regional information in the form of ratios of RBC/TP potentially forming the basis of regional gas exchange mapping of the lungs.^62 63^ Wang *et al* demonstrated that these signals showed weak correlation with qualitative CT and semiquantitative CT scores in a cohort of subjects with IPF but good correlation with PFTs.^64^ Weatherley *et al* recently showed that the ratio of the RBC/TP peaks in patients with IPF is highly sensitive to change when scanned at 0-month, 6-month and 12-month intervals when compared with FVC and DLCO.^65^ These ^129^Xe MRI measures of gas exchange provide a novel means of functional assessment of ILD. Example spectra and dissolved phase imaging are shown in figure 3 and figure 4. However, changes in ^129^Xe MR spectroscopy metrics are likely to result from both interstitial thickening and changes in pulmonary haemodynamics, as a recent dual case report suggests and the influence of each requires further investigation.^66^

Diffusion-weighted MRI of HP gases enables quantification of the Brownian motion of these gases in the acinus by mapping of the apparent diffusion coefficient (ADC) of the gas. The ADC is reflective of acinar airway integrity and microstructural length scales in pulmonary disorders, such as emphysema.^44^ Altered ADC has been reported in a small cohort of patients with pulmonary fibrosis, indicating a disruption of the acinar microstructure, but it is not clear if these changes precede structural changes evident on CT.^43^

### Dynamic contrast-enhanced MRI

**Figure 5:**
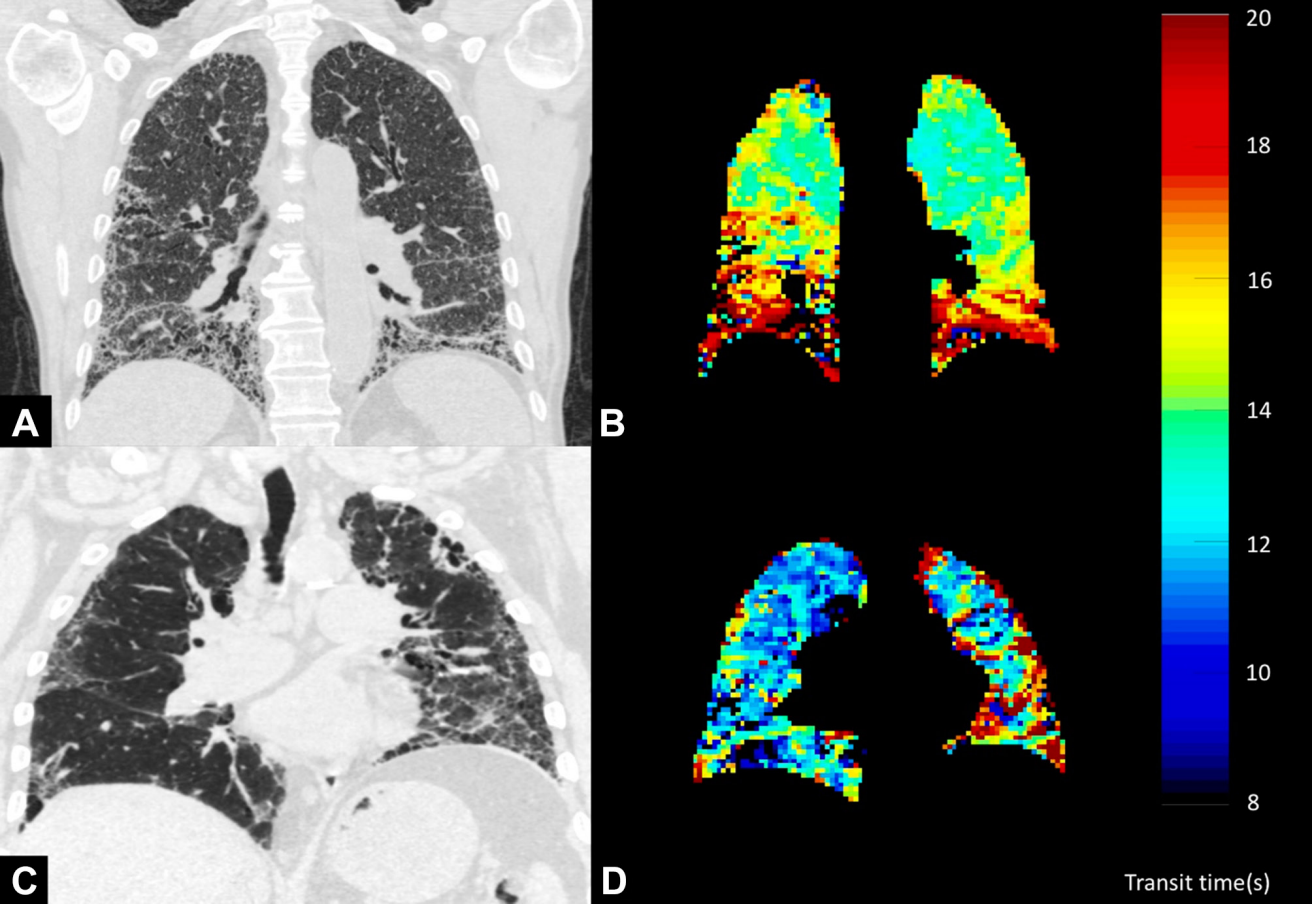
In patients with interstitial lung disease (ILD), transit time of intravenous contrast across the lungs is increased in anatomical regions of interstitial change. The rows (A and B and C and D) represent two patients with ILD. The coronal reconstruction of their CT scans demonstrates ILD, predominantly in both bases in A and predominantly in the left lung periphery in C. Transit times within these voxels in the parametric maps derived from dynamic contrast-enhanced MRI (B and D) demonstrate increases in transit time through these area, suggesting perfusion limitation.

MR contrast uptake parameters using dynamic contrast-enhanced (DCE) MRI provide a means of pulmonary haemodynamic assessment. Tsuchiya *et al* illustrated that pulmonary arterial flow was slower in a cohort of patients with ILD than in a group of volunteers without lung disease.^67^ However, pulmonary arterial flow as a single measure did not demonstrate correlation with disease severity. In a study of patients with CPFE, mean transit time (MTT) through the lung was prolonged compared with healthy controls.^68^ MTT also correlated with pulmonary artery pressure and pulmonary vascular resistance, measured by right heart catheterisation. Example transit time images alongside representative anatomical CT slices in patients with IPF are shown in figure 5. Metrics such as pulmonary blood volume variation during the cardiac cycle may provide early warning of pulmonary haemodynamic changes in ILD or impending pulmonary hypertension.^69^

Early and late contrast enhancement features may also help differentiate inflammation from fibrotic-predominant pathology. Yi and colleagues dichotomised biopsy specimens from 26 patients with UIP into inflammation or fibrosis predominant, finding that early T1 enhancement was more likely to be associated with inflammation.^70^ Mirsadraee *et al* also demonstrated the feasibility of pre-contrast and post-contrast T1 mapping of IPF.^71^ In 10 patients with IPF, contrast uptake was delayed compared with 10 healthy volunteers, including in regions with ‘normal’ precontrast T1 values, suggesting that early perfusion changes may be detectable prior to morphological changes. Another study also demonstrated that late contrast-enhanced MRI signal was significantly increased in the lungs of 20 patients with IPF compared with 12 healthy volunteers.^72^ There was a strong correlation between the degree of pulmonary fibrosis on late enhanced MRI and HRCT.

## MRI summary

Several MRI metrics from conventional, oxygen-enhanced, hyperpolarised gas and DCE MRI are under exploration. A number of promising methods are available; however, assessment of the reproducibility of derived metrics and longitudinal observation in human participants with ILD is required to assess their suitability as accurate markers of disease. Functional MRI metrics will also benefit from direct comparison with structural imaging approaches through image registration techniques. Novel molecularly sensitive MR techniques such as collagen-targeted chelated MR agents,^73^ or acidoCEST MRI for extracellular pH estimation,^74^ may provide further opportunities for novel MRI of ILD in the coming years. At present, this remains a research tool, but use in early phase pharmaceutical intervention programmes can be envisaged in the near future.

### Positron emission tomography

PET is rarely clinically used in ILD. Inflammatory predominant and thus metabolically active ILDs provide the most intuitive targets for the use of ^18^F-fluorodeoxyglucose (FDG) PET. For example, Tateishi *et al* demonstrated that the standardised uptake value (SUV) of FDG correlated with lymphocyte activity in 22 patients with organising pneumonia,^75^ while PET activity in active and persistent pulmonary sarcoidosis is associated with serological evidence of inflammation and loss of pulmonary function.^76^

The role of PET in fibrotic-predominant ILD is less intuitive, and one may expect inflammatory-predominant conditions to be more metabolically active. However, Jacqelin *et al* identified 18 patients with cellular and fibrotic NSIP, finding that consolidation, GGO, honeycombing and reticulation showed uptake in 90%, 89%, 85% and 76% of regions, respectively.^77^ Groves *et al* evidenced that SUV increased in IPF and that ^18^FDG metabolism appeared higher in regions of reticulation and honeycombing when compared with ground glass regions.^78^ Interestingly, there is also evidence of increased FDG uptake in apparently normal lung tissue of patients with ILD identified by visual inspection and CT density analysis, raising the question as to whether SUV is identifying subclinical disease.^79^

Other authors have explored the role of PET in fibrosis-predominant ILD^80^ and reproducibility in IPF.^81^ A small study involving eight patients with IPF showed that over a period of 6 months, the decline in FVC was strongly correlated with an increase in maximal SUV but was not associated with the visual CT score.^82^ Umeda *et al* demonstrated that delayed-phase ^18^FDG uptake may provide a marker of disease activity in idiopathic interstitial pneumonia^83^ and later also specifically in IPF.^84^ Increased late phase uptake was predictive of mortality on both univariate and multivariate analysis. Although the numbers in both studies were relatively small, the IPF cohort was prospectively followed up for a median of 29 months, with 25 deaths.^84^ In a larger cohort of 113 patients, Win and colleagues demonstrated that the target to background ratio, calculated by dividing the maximal SUV by minimal SUV, predicted mortality over a mean 29-month follow-up, independent of GAP score.^85^ Novel PET tracers and targets such as somatostatin receptor analogues,^86^ cathepsin protease (macrophage labelling),^87^ labelled leukocytes^88^ and type I collagen^89^ have shown early promise in ILD assessment and further reports are awaited.

## PET summary

The high SUV seen in normal lung tissue in patients with ILD is intriguing. Further dual-time-point studies would be useful to assess if these areas go on to develop appreciable fibrotic change on structural images, ideally using formal CT registration tools. Novel PET tracers are yet to be clinically assessed and although they are promising, the requirement on a cyclotron for on-site manufacture of these short-lived molecules may inhibit widespread uptake.

### Conclusions

Quantitative analysis of multimodal imaging is likely to play an increasing role for combined pulmonary structure-function assessment in many pulmonary disorders. While these approaches require further external validation and are not yet ready for routine use in clinical practice, they are likely to be additive to ILD assessment and may play an important role in diagnosis and assessing treatment response. Quantifying pulmonary vessel changes may prove to be an outright novel metric in ILD assessment. There is great promise that both supervised and unsupervised approaches applied to large, well-characterised datasets may lead to discovery of additional meaningful features of ILD (radiomics or imaging biomarkers). Clustering techniques and machine learning may allow automated stratification of patients into disease categories and risk groups and provide decision support that may aid in the selection of optimal therapy and a means to assess efficacy. MRI and PET remain exploratory techniques in ILD but may provide a link between functional and anatomical elements of disease, such as is demonstrated by regional gas exchange in^129^Xe MR spectroscopy. Novel PET tracer agents may be used in early drug development programmes and indeed a study using FDG PET avidity to assess response to dabigatran in IPF is currently recruiting to trial (ClinicalTrials.gov identifier NCT02885961).

10.1136/thoraxjnl-2018-211779.supp1 Supplementary data

10.1136/thoraxjnl-2018-211779.supp2 Supplementary data
