# Supplementary material for: Experimental and quantitative imaging techniques in interstitial lung disease
Source: Thorax. 2019 Mar 18;74(6):611–9. doi: 10.1136/thoraxjnl-2018-211779 (PMC6585263; doi:10.1136/thoraxjnl-2018-211779)
Supplement: Supplementary data [file thoraxjnl-2018-211779supp001.pdf]

PRISMA 2009 Flow Diagram

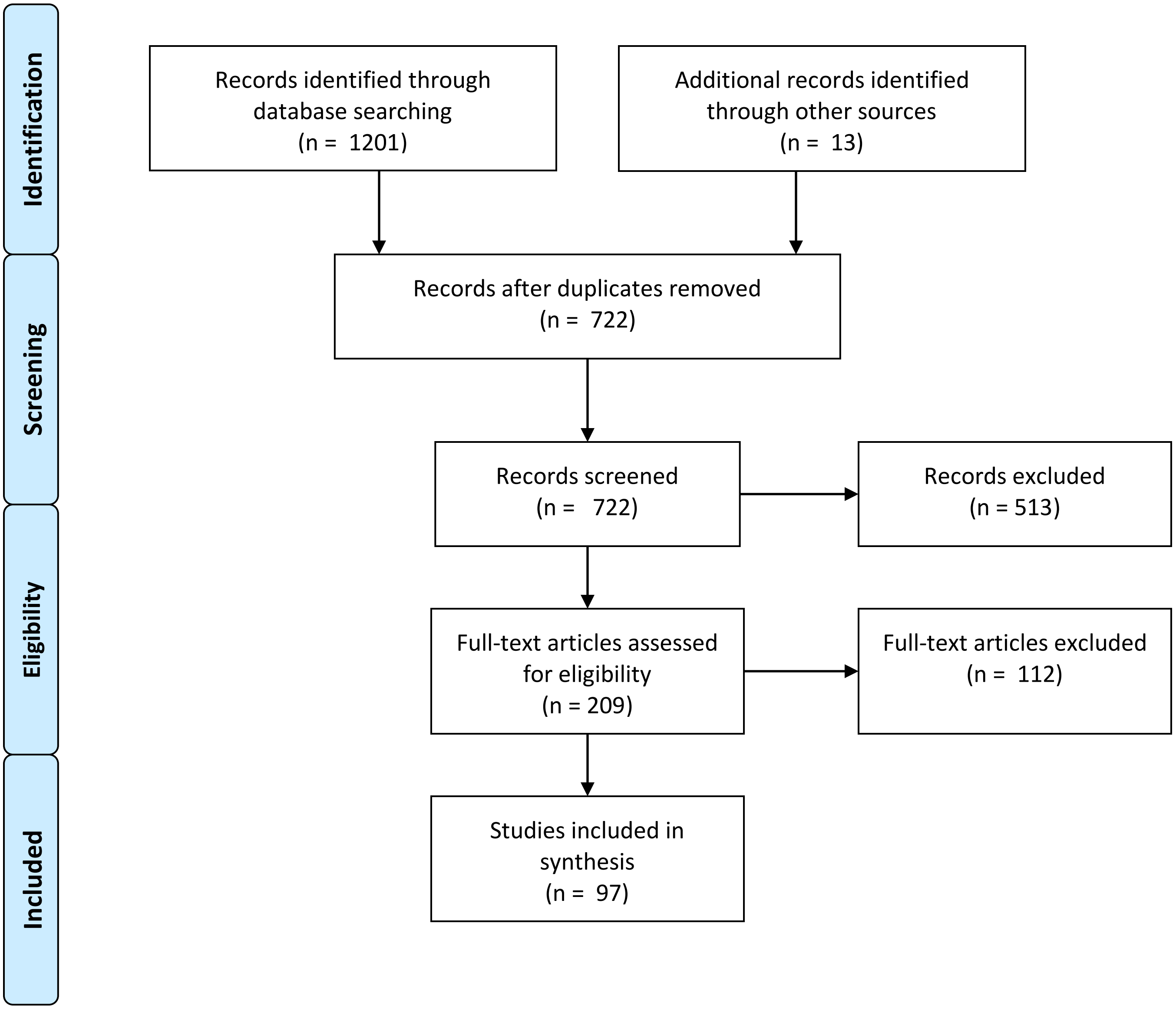

From: Moher D, Liberati A, Tetzlaff J, Altman DG, The PRISMA Group (2009). *P*referred *R*eporting *I*tems for *S*ystematic Reviews and *M*eta-*A*nalyses: The PRISMA Statement. PLoS Med 6(7): e1000097. doi:10.1371/journal.pmed1000097

For more information, visit www.prisma-statement.org.
