# Supplementary material for: Experimental and quantitative imaging techniques in interstitial lung disease
Source: Thorax. 2019 Mar 18;74(6):611–9. doi: 10.1136/thoraxjnl-2018-211779 (PMC6585263; doi:10.1136/thoraxjnl-2018-211779)
Supplement: Supplementary data [file thoraxjnl-2018-211779supp002.pdf]

A state of the art commentary of experimental imaging techniques in interstitial lung disease (ILD). A view to developing imaging tools in drug-induced ILD

Databases: Ovid Medline (University of Sheffield), TRIP database, PubMed online

PICO

P: Any form of interstitial lung disease (diffuse parenchymal lung disease)

I: Quantitative (HR)CT, Any MR(I) modality, PET, SPECT

C: (HR)CT, pulmonary function tests

O: Prognosis, prediction, severity measures, phenotyping

Outcome left open to ensure widest possible data collection

Comparator not specifically supplied for the same reason

Search terms

Three searches carried out:

1. **CT:**

MESH: [Tomography, X-Ray Computed (exp)]

AND

MESH: [Pattern Recognition, Automated (exp)]

OR

Free text: quantitative.mp, automat?.mp. [mp=title, abstract, original title, name of substance word, subject heading word, keyword heading word, protocol supplementary concept word, rare disease supplementary concept word, unique identifier, synonyms], automated.mp.

2. **MRI:**

MESH: [magnetic resonance imaging (exp)], [xenon (exp)], [helium (exp)]

OR

Free text: magnetic resonance.ab.ti. MRI.ab.ti, NMR.ab.ti, oxygen enhance*.ab.ti, xenon, helium, xe.ab.ti, hyperpolari*. ab.ti

3. **PET and SPECT:**

MESH: [Tomography, Emission-Computed (exp) tomography, Emission-Computed, Single-Photon (exp)]

OR

Free text: PET.mp, positron electron tomograph*.mp., SPECT

Each search was combined with the following terms*:*

AND

MESH: [Lung diseases, Interstitial (exp), Pulmonary Fibrosis (exp),]

OR

Free text: Difffuse parenchymal lung*, "pulmonary fibro*.ab,ti., lung fibro*.ab,ti.

Limited to English language

Evaluation of evidence

The article is to be a state of the art review of advances in novel lung imaging to assess interstitial lung diseases. It will provide a narrative review of the recent evidence and as such no formal grading of evidence quality will be undertaken. The analysis will aim to return to the question of how such imaging can be used to assess drug-induced interstitial lung disease.
